# Integration of Mitochondrial Targeting for Molecular Cancer Therapeutics

**DOI:** 10.1155/2015/283145

**Published:** 2015-12-02

**Authors:** Philippe Marchetti, Pierre Guerreschi, Laurent Mortier, Jerome Kluza

**Affiliations:** ^1^Inserm UMR-S 1172, Faculté de Médecine, Université de Lille, 59045 Place Verdun Cedex, France; ^2^Centre Hospitalier Régional et Universitaire (CHRU) de Lille, 5900 Lille, France

## Abstract

Mitochondrial metabolism greatly influences cancer cell survival, invasion, metastasis, and resistance to many anticancer drugs. Furthermore, molecular-targeted therapies (e.g., oncogenic kinase inhibitors) create a dependence of surviving cells on mitochondrial metabolism. For these reasons, inhibition of mitochondrial metabolism represents promising therapeutic pathways in cancer. This review provides an overview of mitochondrial metabolism in cancer and discusses the limitations of mitochondrial inhibition for cancer treatment. Finally, we present preclinical evidence that mitochondrial inhibition could be associated with oncogenic “drivers” inhibitors, which may lead to innovative drug combinations for improving the efficacy of molecular-targeted therapy.

## 1. Introduction

**Figure 1:**
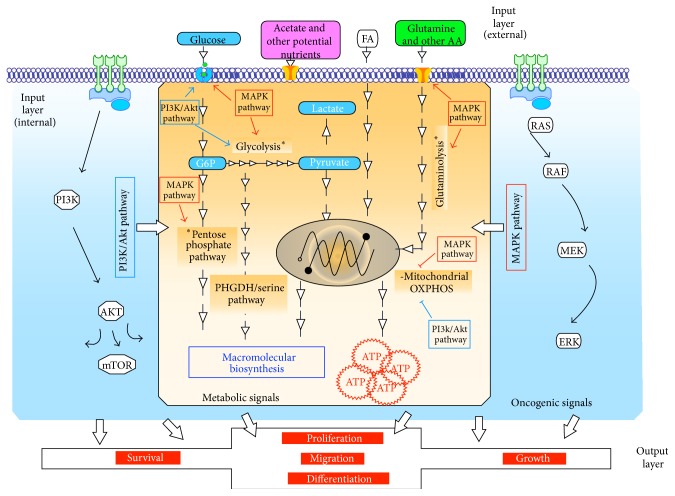
General organization of the metabolic networks in cancer cells. The input layer's internal (oncogenic signals) and external (nutrients in the environment) signals influence the organization of metabolic pathways and thereby regulate the output layer (see text for details). The general impact of the main oncogenic signals (PI3K/Akt and MAPK pathways) on the metabolic organization of cancer cells is illustrated.

**Figure 2:**
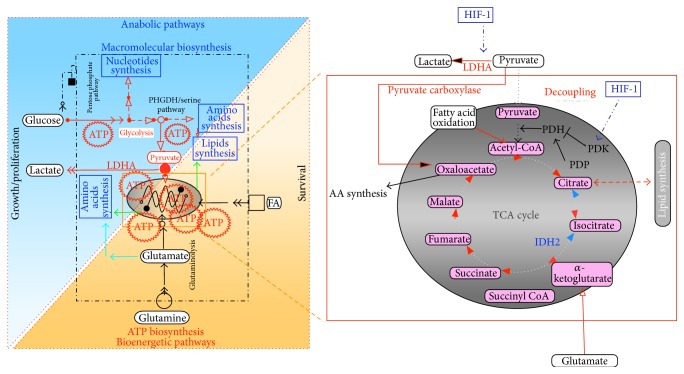
Schematic diagram of metabolic networks placing mitochondria at the center of anabolic and bioenergetics pathways in cancer cells. Anabolic pathways are responsible for the production of macromolecules used for growth and proliferation of cancer cells. Red lines indicate glycolysis: multisteps conversion of glucose to pyruvate and pyruvate to lactate allowing the flux of glucose intermediates to fulfill anabolic pathways such as the pentose phosphate pathway and the PHGDH/serine pathway for nucleotides, lipids, and AA biosynthesis (see text for details). The decoupling of glycolysis from mitochondria is also observed. Mitochondria participate in ATP production through oxidation of alternative substrates such as glutamine or fatty acid (FA). Furthermore, mitochondria are also involved in anabolic pathways for producing building blocks (AA, lipids). Glutamine refills TCA intermediates (anaplerosis) and can feed the reverse TCA cycle for lipid synthesis (blue arrows) (see text for details).

Over the past decades, intensive research has emerged to complete and further understand the initial observations of Warburg on cancer cell metabolism (for review [1]). According to Warburg [2], cancer cells reprogram their metabolism into intense glycolysis regardless of oxygen presence, a phenomenon also known as aerobic glycolysis. The so-called “Warburg phenotype” compromises high glucose uptake followed by high level of glycolytic activity producing pyruvate decoupled from mitochondrial oxidation, which undergoes fermentation into lactic acid (Figures 1 and 2). The stepwise cytoplasmic breakdown of glucose generates several glycolytic intermediates that feed preferentially alternative anabolic pathways, thus allowing the biosynthesis of building blocks promoting rapid cellular proliferation. Glycolysis-derived anabolic pathways include the pentose phosphate pathway for* de novo* biosynthesis of nucleic acid and the phosphoglycerate dehydrogenase/serine pathway for AA synthesis and/or lipid synthesis (Figure 1).

Cellular metabolism, which reflects the integration of several signals from multiple coordinated pathways, is a context-dependent process (dependent on tissue type and oncogenic influence) tightly linked to cellular fate [3]. The reprogramming of cancer cell metabolism results from both environmental signals (external input), such as oxygen level or access to nutrient, and oncogenic pathways (internal input), to make up a network of input layers (Figure 1). As a result, cancer cell metabolism critically influences cellular fate (output layer) such as survival, growth, migration, differentiation, or proliferation (Figure 1). As recently reviewed [1], aberrant stimulation of prominent oncogenic signaling pathways such as the MAPK pathway increases glucose uptake and actively reroutes metabolism into glycolysis, thus providing the needed fuel and building blocks for cell survival and proliferation. These observations indicate that cancer cell metabolism constitutes one part of the aberrant oncogene-driven signaling resulting in the anarchic proliferation of cancer cells. The Warburg phenotype was originally associated with weak mitochondrial activity [4]. Indeed, as a consequence of the intense reduction of pyruvate into lactate, pyruvate is less available for oxidation in the mitochondrial matrix. Nevertheless, accumulated data prove that cancer cell mitochondria are still actively powered, essentially by alternative carbon substrates including glutamine and/or fatty acids (Figure 1) [1]. Moreover, emerging data indicate that the role of cancer cell mitochondria is not restricted to ATP biosynthesis (catabolic pathways) but also encompasses macromolecular biosynthesis (anabolic pathways) (Figure 2). Thus, rather more than initially expected, mitochondrial metabolism plays a key role in cancer cell survival and development.

Given this crucial role of mitochondria at the core of cancer cell fate, the potential to interfere with mitochondrial functions has become a promising source of new targets for anticancer treatment. In this context, this review describes the promises and hurdles of targeting mitochondrial metabolism in cancer and discusses the advantages of integrating this innovative approach to current treatments such as molecular-targeted therapies.

## 2. Characterization of Mitochondrial Metabolism in Cancer Cells 

The important feature of cancer cell metabolism is the low rate of glucose-derived pyruvate, which oxidizes in the mitochondria resulting in a decoupling of the mitochondrial glycolytic flux (Figure 2). This is mainly due to the inactivation of the gatekeeper enzymatic complex, pyruvate dehydrogenase (PDH), responsible for the entrance of pyruvate into the mitochondria. Its enzymatic activity is tightly dependent on the reversible phosphorylation of serine residues. Phosphorylated PDH by PDK enzymes (PDK1–4 isoenzymes) is inactive; conversely, PDH dephosphorylation by PDP1 and PDP2 enzymes stimulates PDH activity as well as the oxidation of pyruvate in the mitochondria. Interestingly, this checkpoint is controlled by HIF-1*α*. PDKs are direct transcriptional targets of HIF-1*α*, the major factor controlling cellular responses to hypoxia. Thus, HIF-1*α* blocks the pyruvate flux from entering the mitochondria via its inhibitory effect on PDH [5]. HIF-1*α* also promotes LDHA expression, the enzyme responsible for the degradation of pyruvate into lactate. This HIF-1*α*-dependent effect on LDHA shunts the pyruvate flux away from mitochondrial oxidation. Since glycolysis is decoupled from the mitochondrial metabolism via PDH inactivation, cancer cell mitochondria have to circumvent the PDH inactivation to maintain their functions. Cancer cells use two main pathways to sustain mitochondrial activity: (i) glucose-derived pyruvate can undergo irreversible carboxylation and form the mitochondrial TCA intermediates, that is, oxaloacetate. This conversion is catalyzed by pyruvate carboxylase, a mitochondrial biosynthetic enzyme particularly important in cancer [6]. (ii) In the absence of available glucose-derived carbon, cancer cell mitochondria can also use fatty acids or glutamine (the most abundant amino acid in humans) as an alternative carbon source. Many cancer cell types (including melanoma [7], glioblastoma [8], and leukemia [9]) depend on glutamine metabolism for survival, growth, and proliferation. Thus, *α*-ketoglutarate derived from glutamine represents the major fuel source for the TCA cycle under hypoxia [7]. Glutamine uptake and use are critically controlled by key oncogenes including c-Myc or Ras. Mitochondrial metabolism supported by glutamine is required for KRAS-dependent tumorigenicity [10]. Glutamine also supports the proliferation of malignant cells through a reductive IDH-dependent TCA pathway (reverse direction) even when mitochondrial oxidative metabolism is defective [11]. The decoupling of glycolytic flux from mitochondria allows the mitochondria to function in anabolic mode using glutamine as an anaplerotic substrate [12]. This could be explained by the interrelationship between both pyruvate metabolism and glutamine metabolism since glutamine oxidation (glutaminolysis) depends on the availability of pyruvate for transamination. Thus, activation of mitochondrial PDH impairs glutamine metabolism and subsequently alters cell growth [13]. This illustrates the existence of a subtle balance between glucose and glutamine in mitochondrial use. In addition to these carbon sources, fatty acid is a relevant “feeder” for supporting mitochondrial activity in cancer, providing the extra “ATP” required for survival [14]. Finally, there are also less common alternate substrates such as lactate [15] maintaining mitochondrial activity when glucose, glutamine, and/or fatty acid are unavailable (see below). Overall, cancer cell mitochondria can metabolize a large variety of carbon substrates according to nutrient availability and oncogenic signals and can guide cellular fate as well as modifying most cellular functions.

## 3. Why Is It Attractive to Target Mitochondrial Metabolism in Cancer? 

Nowadays, mitochondrial metabolism is currently recognized as a potential source of targets for anticancer agents due to the metabolic peculiarities of cancer cells. As mentioned above, the rationale of mitochondria-based strategies comes from the convincing demonstration that mitochondrial metabolism is a key player in cancer development and progression [43–47]. Indeed, evidence supporting the role of mitochondria in cancer is summarized as follows.

*Firstly, Mitochondrial Activity Contributes to Cancer Cell Survival.* Given the decoupling of glycolytic flux from mitochondria, mitochondrial glutaminolysis is preferentially used to produce ATP contributing to supporting cancer cell survival [8]. Glutamine is crucial for the development of BRAF mutated (such as BRAF^V600E^) lung tumors [48]. Interestingly, autophagy (self-eating) is an essential source of glutamine for mitochondrial metabolism [48]. Thus, autophagy-deficient BRAF^V600E^ tumors present a significantly impaired mitochondrial respiration leading to a subsequent decrease in cell survival, which can be rescued by the addition of exogenous glutamine [48]. As mentioned above, oxidation of alternative substrates such as FA can participate to mitochondrial ATP production and cell survival [14]. Apart from its role in ATP production, mitochondrial metabolism allows for the generation of reactive oxygen species (ROS) which are also crucial for tumor cell survival and development [10].

*Secondly, Mitochondrial Activity Promotes Cell Invasion and Metastasis*. Whereas the major function of glucose metabolism is to support growth (e.g., via the pentose phosphate pathway), KRAS-mutated colon cancers require mitochondrial glutamine metabolism for anchorage-independent growth [10]. Invasive and metastatic cancer cells rely mainly on mitochondrial oxidative phosphorylation (OXPHOS) which is activated by the peroxisome-proliferator-activated receptor coactivator-1*α* (PGC-1*α*), a crucial transcriptional regulator for mitochondrial biogenesis and function [49]. Enforced PGC-1*α* expression promoting invasion, and conversely the formation of lung metastasis, is significantly impaired when PGC-1*α* expression is inhibited [5, 49]. Likewise, mitochondrial activity is significantly correlated to the invasive potential of cancer cells [46]. This observation can be explained by the fact that the overproduction of mitochondrial ROS, resulting from intense mitochondrial activity, activates the protein tyrosine kinases Src and Pyk2, which, in turn, promotes carcinoma invasion [46]. Besides, migratory cancer cells depend on mitochondria for ATP production, an energy source required for survival in conditions imposed by metastatic colonization [50, 51]. Moreover, cancer cells without mitochondrial DNA (*mt*DNA) injected in recipient mice show delayed tumor growth and progression. Intriguingly, some of these cells can acquire* mt*DNA of host origin, resulting in stepwise recovery of mitochondrial functions. Only* mt*DNA-depleted cancer cells capable of recovering mitochondrial activity can metastasize* in vivo* confirming the crucial need of OXPHOS for tumor growth and progression [52].

*Thirdly, Mitochondrial Activity Is Associated with Anticancer Drug Resistance*. Genotoxic drugs induce a shift in cancer metabolism inducing mitochondrial dependency, that is, mitochondrial addiction (characterized by OXPHOS upregulation and mitochondrial biogenesis), which persists in chemotherapy-resistant colorectal tumors [53]. This mitochondrial “boost” is mediated by the activation of the histone deacetylase sirtuin-1 (SIRT1) and its substrate, the coactivator PGC-1*α* [53].

Mitochondrial OXPHOS is also associated with* de novo* and acquired resistance to inhibitors of oncogenic kinases including MAPK inhibitors [19, 35, 45]. Thus, BRAF and NRAS mutant melanomas contain a subpopulation of cells intrinsically resistant to MEK inhibitors, which displays a classic OXPHOS phenotype and PGC1*α*-dependent mitochondrial biogenesis [35]. It is noteworthy that melanoma cells with acquired resistance to BRAF inhibitors (BRAFi) maintain an OXPHOS phenotype regardless of the underlying resistance mechanism [19]. This metabolic shift towards oxidative metabolism partly relies on the PGC1*α*-dependent mitochondrial biogenesis. Interestingly, melanomas exposed to BRAFi lead to the enrichment of a drug-tolerant subpopulation of slow-cycling persistent cells. These resistant cells are characterized by the expression of the H3K4 demethylase, JARID1B. The “stem cell-like” JARID1B^high^ subpopulation is addicted to mitochondrial OXPHOS for survival [41]. Likewise, in pancreatic cancer, KRAS ablation selects a subpopulation of “dormant” surviving cells responsible for tumor relapse, identified by a mitochondrial metabolic fingerprint [37]. One can assume that mitochondrial OXPHOS represents more than the metabolic signature of BRAFi-resistant cells. Mitochondrial reprogramming may be seen as an active adaptive phenomenon to BRAFi, which is responsible for the survival of a BRAFi-tolerant cell subpopulation and eventually for the development of an acquired resistance by giving cancer cells the time to accumulate additional mutations (Figure 4 and see Section 6). This reliance of a drug-tolerant subpopulation on mitochondrial activity (mitochondrial addiction) suggests the existence of a potential metabolic breach that could be exploited on a therapeutic level.

*Fourthly, Mitochondria Can Fulfill an Anabolic Role Contributing to Cancer Cell Proliferation.* Apart from glutamine catabolism, other atypical pathways may be used by cancer cells to maintain anabolism in “unfavorable environments” (i.e., with less access to nutrients). As a matter of fact, a recent analysis of tumor metabolomics indicates that cancer cells can oxidize glucose-derived pyruvate in mitochondria via the PDH-dependent pathway supporting the production of glutamine, which is mandatory for tumor growth [54]. This very relevant study using an orthotopic model of human glioblastoma illustrates the complex context-dependent regulation of mitochondrial metabolism in cancer.

Overall, these reports provide convincing evidence supporting the involvement of mitochondria in cancer development and a strong rationale for developing mitochondria-targeted agents to fight cancer.

## 4. What are the Types of Mitochondrial Targets for Cancer Therapy?

**Figure 3:**
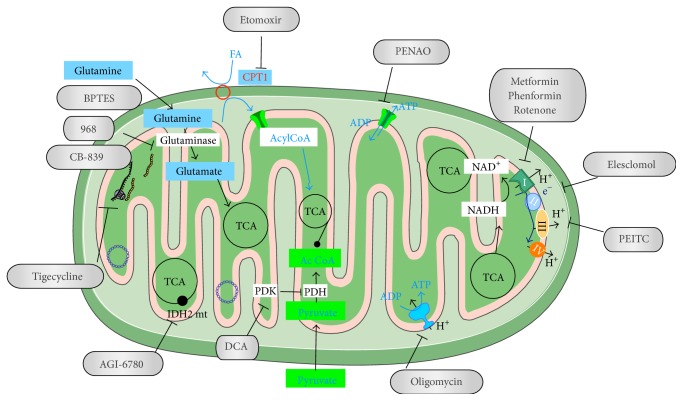
Diagram presenting the main potential mitochondrial targets for cancer treatment (see text for details).

Based on the aforementioned elements, several drugs have been used to directly target mitochondria for inducing cancer cell death (see Table 1 and Figure 3). Among them, elesclomol is a promising investigational drug, currently under clinical trials as a novel anticancer therapeutic. Elesclomol displays potent anticancer activity through the inhibition of the electron transport chain. As a consequence of elesclomol exposure, cells block ATP production, promoting mitochondrial ROS generation and finally cell death. Interestingly, elesclomol preferentially binds extracellular copper and selectively transports this metal ion to the tumor cell mitochondria reducing its adverse toxicity in normal tissues [19, 55]. Conversely, the activation of mitochondrial metabolism via increased pyruvate oxidation in the mitochondrial matrix (e.g., by inhibiting the gatekeeper PDK enzymes or LDHA) has also been shown to impede cancer development [5, 56]. This latter approach seems clinically feasible since “the mitochondrial booster” dichloroacetate (DCA), a small-molecule PDK inhibitor, has been previously prescribed for several years in mitochondrial diseases without exhibiting major side effects [57]. DCA by PDH activation redirects pyruvate metabolism back into the mitochondria and then increases mitochondrial functions resulting in a strong reduction in anabolic glycolysis (reversing the Warburg effect) and, therefore, in cell proliferation [58]. Moreover, DCA overproduces cytotoxic ROS, as a by-product of mitochondrial OXPHOS, thereby promoting cancer cell death [5]. However, despite promising preclinical data, DCA remains an experimental anticancer treatment, which lacks robust clinical information to become clinically approved.

The most promising therapeutic approach to target glutamine catabolism stems from the inhibition of glutaminase. Two glutaminase inhibitors 968 and BPTES [*bis*-2-(5-phenyl-acetamido-1,2,4-thiadiazol-2-yl)ethyl sulfide] have demonstrated antitumor effects in xenograft studies. Interestingly, CB-839, a selective orally bioavailable inhibitor of human glutaminase, is currently in clinical trials. The use of other classical inhibitors of glutamine metabolism such as the 6-diazo-5-oxo-L-norleucine (DON) is limited by toxicity.

Classical inhibitors of mitochondrial OXPHOS (e.g., the complex V inhibitor, oligomycin, or the complex I inhibitor, rotenone) appear to be challenging for clinical implementation; their absence of specificity and dosage requirements limit their use for anticancer treatment. Since most of these drugs are not yet translatable to human clinical trials, new strategies to improve the therapeutic activity of mitochondrial drugs are currently under development [59].

Due to these current limitations, other efforts focused on more indirect approaches to block signals upstream mitochondria. Oxygenated tumors are able to metabolize lactate as the preferential substrate for mitochondrial OXPHOS [15]. Thus, the inhibition of the monocarboxylate transporter 1 (MCT1), main lactate importer in cancer cells, blocks lactate-dependent mitochondrial respiration and therefore defines MCT1 as a potential anticancer target [15].

## 5. Current Challenges in Mitochondrial Targeting

In these past years, there has been an emergence of new data on molecular and biological regulation of cancer metabolism. These insights have changed the comprehension of the role played by metabolism in cancer. Importantly, these considerations (listed below) should be taken into account when considering mitochondrial targeting for cancer treatment.

### 5.1. Mitochondrial Metabolic Heterogeneity of Cancer

Several lines of evidence indicate the existence of inter- and intratumor differences in mitochondrial metabolism [60, 61]. Mitochondrial activity may vary depending on additional intra- and extracellular factors and is not always associated with metastasis. Colon cancer metastasis has been associated with cell selection characterized by Warburg's phenotype, that is, high glycolysis decoupled from mitochondrial oxidation [62]. Likewise, the mitochondrial pyruvate complex (MPC), which ensures efficient mitochondrial pyruvate uptake, is downregulated in some colon cancer types and its low expression is correlated with poor prognosis. The reexpression of MPC reduces anchorage-independent growth* in vitro* and decreases the expression of colon cancer stem cell markers [63].

Mitochondrial metabolic heterogeneity can be explained by a variety of reasons including the genetic background of cancer cells, nutrient availability, and cell fate (Figure 1).

*Firstly, Mitochondrial Metabolic Heterogeneity Reflects the Genetic Heterogeneity of Tumors*. Somatic or germline mutations in mitochondrial metabolic enzymes have been found to be causally involved in tumorigenesis [64]. Mutations in the isocitrate dehydrogenase 2 (IDH2) genes have been mainly reported in acute myeloid leukemia and glioblastoma. Loss of fumarate hydratase (FH) function has been associated with the development of hereditary leiomyomas and renal cell carcinoma, whereas succinate dehydrogenase (SDH) mutations account for paragangliomas and pheochromocytomas. Cancer cells that contain these mutants can survive without a functional TCA cycle. Furthermore, TP53, inactivated in more than 50% of solid cancers, regulates mitochondrial respiration. TP53 induces the expression of the mitochondrial metallochaperone protein SCO2, which is required for cytochrome c oxidase (complex IV) assembly and ETC effectiveness [65].

Apart from these genetic alterations, oncogenic driver mutations can also affect the mitochondrial function. The mutant BRAF, BRAF^V600E^, reprograms cancer cell metabolism from OXPHOS to aerobic glycolysis. The expression of BRAF^V600E^ is correlated with high glucose uptake and the expression of the transporter GLUT-1 in different types of cancer (for review [1]). In melanoma, oncogenic BRAF^V600E^ promotes the mRNA and protein expression of GLUT-1, GLUT-3, and HK2 through the involvement of several transcription factors including HIF-1*α*, c-Myc, and MONDOA [50]. Importantly, the expression of GLUT-1, GLUT-3, and HK2 is reduced in melanoma specimen from patients treated by pharmacological BRAF^V600E^ inhibitors (BRAFi) and is reexpressed after relapse suggesting a critical role of glycolysis in melanoma progression. A subpopulation of melanoma cells expresses the melanocyte lineage-specific transcription factor MITF (microphthalmia-associated transcription factor), which upregulates PGC-1*α*, resulting in mitochondrial biogenesis increase and therefore rendering cells addicted to mitochondrial activity [45, 47]. Besides, MITF is a downstream target of BRAF^V600E^. Thus, constitutive activation of the oncogenic mutant BRAF^V600E^, occurring in 50% of melanomas, represses the MITF/PGC-1*α* axis and, in turn, lowers mitochondrial OXPHOS [45]. Since the genetic signature determines the metabolic network and can explain intertumors metabolic heterogeneity, the genetic background could predict mitochondrial activity in cancer.

*Secondly, Mitochondrial Metabolism Depends on the Availability of Oxygen (and Nutrients) in the Environment*. During tumor growth, the anarchic formation of blood vessels results in heterogeneous distribution of oxygen with areas of normoxia and hypoxia within the tumor. PET Scan analysis of FDG uptake unveils high levels of metabolic heterogeneity within tumors [66]. Mitochondrial metabolism is directly related to the distance of the cancer cells from the blood vessels [67]. Within solid tumors, well-oxygenated (aerobic) and poor-oxygenated (hypoxic) regions coexist; they contain cells using, respectively, oxidative (mitochondrial) and glycolytic metabolisms.

Mitochondria of cancer cells enable metabolizing metabolic “waste” including pyruvate derived from exogenous lactate [15] or acetate [68, 69] to compensate nutrient deprivation. These possibilities allow cells to maintain a mitochondrial activity and thereby render cancer cells resistant to metabolic stress. Likewise, glutamine or lipids can be a substitute for glucose and are used by mitochondria to facilitate cell survival, growth, and proliferation. Thus, inhibition of the glycolytic pathway through LDH downregulation [56] or HIF-1*α* knockdown [5] reprograms cancer cell metabolism towards mitochondrial activities. Interestingly, the existence of a “metabolic cooperativity or metabolic symbiosis” between cancer cells, the extracellular space, and the nontransformed neighbor cells has been suspected. According to this model, hypoxic cancer cells or fibroblasts exhibit a glycolytic phenotype since they consume high quantities of glucose and produce high levels of lactate, carried in the microenvironment via the monocarboxylate transporter 4 (MCT-4). Conversely, the extracellular lactate, the waste by-product of hypoxic cells, can be metabolized by oxygenated cancer cells after importation of lactate into cells by MCT-1 and then converted back to pyruvate which in turn is oxidized into the mitochondria [70].

*Thirdly, Mitochondrial Metabolism Is Also Influenced by Cell Fate and Functions*. Cell metabolism can be seen as multiple connections that integrate extracellular nutrients (external input) and genetic background (internal input) to orientate cell fate outcomes (output) such as growth, proliferation, invasion, or differentiation (Figure 1). In other words, cancer cells develop a metabolic program able to use the substrates available in the most efficient manner to control cell fate and functions. Thus, metabolic properties of primary tumors must be different from those of metastatic cells, given the fact that the latter are mainly energy-demanding (e.g., for invasion) whereas primary tumors rely on anabolic reactions for rapid proliferation. In a murine model used to study simultaneously primary mammary tumors, circulating cancer cells, and lung metastases, the authors demonstrated that each subpopulation relies on specific metabolic circuitries [49]. Specifically, invasive cells exhibit a mitochondrial oxidative phenotype and the suppression of PGC-1*α*-dependent mitochondrial biogenesis prevents invasive and metastatic capacities [49]. Metabolic heterogeneity is a major obstacle for using effective mitochondrial inhibitors for anticancer treatment. This heterogeneity underlines the need for potential functional, genetic, and/or phenotypic biomarkers able to predict the response to mitochondrial inhibitors (for review, see [71]).

### 5.2. Metabolic Plasticity of Cancer Cells

Cancer cells possess the ability to adapt their metabolism dynamically in order to maintain growth, survival, and a high proliferative rate even within a hostile environment characterized by hypoxia and limited access to nutrients. As an example, melanoma metabolism has been found to be highly flexible with the ability to adapt to nutrient fluctuations [72]. This characteristic is originally illustrated by the Warburg phenotype, which corresponds to the HIF-1*α*-dependent switch from oxidative to glycolytic metabolism allowing cancer cells to survive with reduced O_2_ availability. This adaptability relies on the subtle balance in mitochondrial use of glycolysis and glutamine, one compensating for the other to sustain mitochondrial activity. Blocking glucose-derived pyruvate oxidation in mitochondria renders tumor cells dependent on the mitochondrial use of glutamine. Conversely, activating the mitochondrial gatekeeper, pyruvate dehydrogenase, which increases the oxidation of pyruvate in the mitochondria, renders cells independent of glutaminolysis [5]. Another example of adaptability is observed with the downexpression of the mitochondrial pyruvate carrier (MPC), which blocks the use of pyruvate in mitochondria, allowing the recourse to other substrates including glutamine,* de novo* lipogenesis, and branched chain amino acids to maintain anabolic and catabolic reactions in mitochondria [73]. Similarly, the mitochondrial protein UCP2 drives the choice of mitochondrial substrate. Mitochondria from cancer cells overexpressing UCP2 do not oxidize pyruvate but rather adapt their mitochondrial metabolism by using nonglucose carbon sources such as fatty acid and glutamine [74–76]. Conversely, in glutamine-deficient cells, a compensatory increase in pyruvate carboxylate allows mitochondria to use glucose-derived pyruvate for anaplerotic reactions [77]. Interestingly, leukemic stem cells, unlike leukemic blasts, lack metabolic flexibility. Indeed, mitochondrial inhibition is not correctly compensated by the increased glycolysis suggesting the existence of a possible metabolic vulnerability of leukemia stem cells [78].

Besides, it has been admitted that the metabolic flexibility of cancer cell could compromise the apoptosis efficacy of mitochondria-targeted drugs. Inhibition of OXPHOS by the complex V inhibitor, oligomycin, activates the metabolic sensor, AMPK, and then shifts the bioenergetics metabolism towards glycolysis and favors survival [79]. Likewise, inhibition of the mitochondrial electron transport chain by the preclinical drug elesclomol can induce, in survival cells, a compensatory glycolysis increase [20]. Furthermore, studies showed mitochondrial biogenesis inhibition via the suppression of the transcription cofactor PGC-1*α* triggers the emergence of a metabolic compensation state promoting melanoma survival and development. The compensatory pathways encompass a ROS-dependent activation of HIF-1*α* leading to high levels of glycolysis followed by a high dependence on glutamine use for melanoma growth and survival [72].

All these elements indicate that tumor cells display dynamic capacities for metabolic adaptation enabling them to switch from one metabolic program to another, limiting the efficacy of mitochondrial targeting for anticancer treatment.

### 5.3. Lack of Specificity of Mitochondrial Metabolism in Cancer

Most of the aforementioned characteristics of cancer metabolism, including mitochondrial heterogeneity and flexibility, are also important features of metabolism in nontransformed cells [80, 81]. In regard to cancer cells, the metabolism of nontransformed cells has to be highly flexible to adapt to nutrient and energy variations. In the context of a fasting diet, muscle cells and liver cells are able to rely on fatty acids instead of glucose as an energy source. Very similarly, metabolic pathways (e.g., Warburg's phenotype) define not only proliferative cancer cells but also other proliferating nontransformed cells including activated lymphocytes (for review [82]). The concept of metabolic symbiosis in tumors, that is, a dialog between anaerobic and aerobic tumor cells via the lactate shuttle (see above), was previously demonstrated in the human brain for neurons and astrocytes [83].

Since metabolic pathways organized in cancer cells also participate in the normal physiological process, the main limitation of the general mitochondrial metabolism inhibition might be the lack of specificity of this approach in cancer treatment resulting in the development of unwanted adverse effects.

## 6. Reprogramming Mitochondrial Metabolism via the Selective Inhibition of Oncogenic Kinases and Its Influence on the Therapeutic Responses of Targeted Therapies

### 6.1. Exposure to MAPK Inhibitors Inhibits Glycolysis and Induces Subsequent Cell Death by Apoptosis

One interesting relationship exists between mitochondrial metabolism and the cellular response to targeted therapies. Targeted therapies can induce deep metabolic changes that regulate treatment response. These metabolic effects were recently described in detail in the context of melanoma exposed to BRAF mutated inhibitors. The MAPK pathway plays a key role in driving aerobic glycolysis and therefore it is not surprising to observe that the inhibition of mutated BRAF or MEK leads to the reduction of glucose uptake and glycolysis. Thus, exposure of BRAF^V600E^ mutant melanoma cells to BRAFi substantially decreases the expression of glucose transporter proteins (GLUT 1, GLUT 3) as well as the expression of hexokinase II, the main rate-limiting enzyme of glycolysis, contributing to reduced extracellular lactate levels [50]. This decreased glucose metabolism has been observed* in vitro* and* in vivo* as FDG uptake is significantly decreased in several animal models exposed to BRAFi [84, 85].

**Figure 4:**
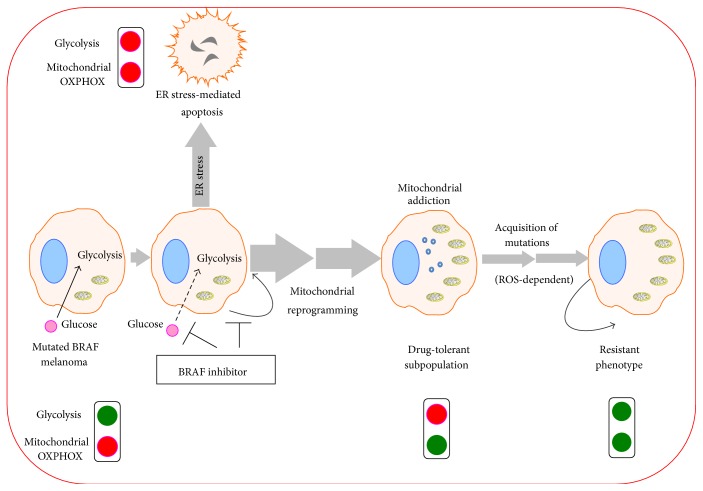
Hypothetical diagram depicting roles of mitochondrial reprogramming in BRAF mutated cells when exposed to BRAF inhibitors (see text for details). Mutated BRAF melanoma mainly relies on aerobic glycolysis. Upon BRAFi exposure, glucose uptake and glycolysis are inhibited leading to ER stress and cell death by apoptosis and consequent energetic collapse (inhibition of both glycolysis and mitochondrial OXPHOS). However, there remains a subpopulation of BRAFi-tolerant cells. These cells reprogram the metabolism towards mitochondrial oxidation in order to survive and consequently this BRAFi-tolerant subpopulation of cells becomes addicted to mitochondria. These surviving cells are prone to accumulating subsequent mutations (potentially induced by mitochondrial ROS overproduction) leading to the onset of a resistant phenotype characterized by aerobic glycolysis associated with high levels of mitochondrial activity (red blot: inhibition, green blot: activation).

Interestingly, the metabolic effects of BRAFi occur before inhibited proliferation [86] suggesting that primarily changes in metabolism could contribute to stopping intense cell proliferation. Unexpectedly, inhibition of oncogenic BRAF^V600E^ does not reactivate the energy sensor, AMPK [87], and does not result in a severe decrease of energy production (personal data) suggesting the development of a metabolic compensation state after BRAF inhibition. Exposure of BRAF mutated melanoma cells to clinically relevant doses of BRAFi leads to apoptotic cell death mediated by ER stress [88]. One can speculate that the early inhibition of glycolysis induced by BRAFi contributes to ER stress and subsequent apoptosis (Figure 4).

### 6.2. Exposure to MAPK Inhibitors Creates a Mitochondrial Addiction for Surviving Cells

The most noticeable feature of cell death induced by clinically relevant doses of BRAFi is its onset at a later stage of exposure (within 72 h) and its moderate rate (<50%). In these conditions, BRAFi exposure appears insufficient to eliminate the overall targeted cell population leaving alive a significant amount of BRAFi-tolerant subpopulation of cells. Consequently, in order to survive in the presence of BRAFi, these cells have to compensate for glycolysis inhibition (Figure 4). If glucose metabolism is disrupted by BRAF inhibition, the BRAFi-surviving cells have to switch to mitochondrial oxidation to maintain an energy-dependent survival. This compensatory state has been described as a mitochondrial addiction since these cells are critically dependent on mitochondrial metabolism for survival. Thus, BRAF mutated cells respond to BRAFi by increasing the mitochondrial membrane potential (Δ*ψ*m), basal and maximal oxygen consumption rates alongside the dynamin-related protein 1 (DRP1) regulated fusion of mitochondria [19, 45, 89]. Gene-set enrichment analysis based on patients' data defines mitochondrial OXPHOS as the metabolic fingerprint in patients treated with BRAFi [45]. Several potential mechanisms resulting in BRAFi-induced mitochondrial reprogramming could be proposed: (i) mobilization of mitochondrial biogenesis through reactivation of the MITF-PGC-1*α* pathway [45, 47], which appears to also be controlled by mTORC1/2 [35]; (ii) inhibition of the HIF-1*α*/PDK pathway [5, 50], the major gatekeeper of mitochondrial activity in melanoma [90]; and (iii) decreased HK2 expression [50], which usually contributes to inhibiting mitochondrial OXPHOS [91]. Likewise, MEK inhibition increases mRNA levels of the transcriptional coactivator PGC-1*α* and MEK inhibitors increase mitochondrial OXPHOS in a PGC-1*α*-dependent manner [35]. Mitochondrial metabolism reprogramming was also observed upon exposure to other driver kinase inhibitors. At high levels (*μ*molar range), the BCR/ABL inhibitor, imatinib, inhibits both glycolysis and mitochondrial activity leading to leukemic cell death [92]. Conversely, sublethal doses of imatinib reduce glucose uptake and glycolysis resulting in a metabolic compensation characterized by an increased TCA cycle and promotion of glutamate synthesis [93, 94].

### 6.3. Mitochondrial Addiction and Therapeutic Escape

As mentioned above (see Section 2), mitochondrial reprogramming is a classic feature of* de novo* cells resistant to anticancer drugs including MAPK inhibitors. Treatment of melanoma cells with BRAFi leads to the enrichment of the JARID subpopulation of slow-cycling melanoma cells characterized by its addiction to OXPHOS [41]. Elevated OXPHOS persists in cell lines and in patients with acquired resistance to BRAF inhibitors, regardless of the resistance-related molecular mechanisms [19, 35]. High levels of PGC-1*α* were correlated with poor prognosis in patients [45]. Elevated PGC-1*α* expression was detected in relapsing tumors upon exposure to MAPK inhibitors [35]. Furthermore, overexpression of PGC-1*α* in BRAF mutated cells alters their sensitivity to BRAFi growth inhibition [40]. Altogether, these results indicate that the mitochondrial reprogramming induced by MAPK inhibitors defines a metabolic state associated with therapeutic escape. One can speculate that the BRAFi-induced mitochondrial addiction allows the development of additional mutations in surviving cells, thus participating in the onset of treatment resistance (Figure 4). While molecular mechanisms need to be more thoroughly refined, the increased mitochondrial OXPHOS induced by BRAFi leads to an overproduction of ROS [19] that could play a role in the development of additional mutations contributing to the reactivation of the MAPK pathway (Figure 4). Likewise, inhibition of glycolysis has been involved in the apparition of MAPK mutations [95], probably through ROS-dependent mechanisms. Overall, oncogenic kinase inhibitors shift cancer cells from oncogene addiction to metabolic (mitochondrial) addiction, which could be involved in the development of treatment resistance (Figure 4).

## 7. Mitochondrial Targeting for Cancer Treatment: New Horizons to Overcome Metabolic Challenges 

Mitochondrial targeting offers attractive opportunities for cancer therapy. However, inhibition of mitochondrial metabolism may activate compensatory pathways, which could still maintain tumor growth and survival. A new strategy to increase anticancer treatment efficacy is to combine mitochondrial targeting drugs with inhibitors of the compensatory metabolic pathways thereby creating an “antimetabolic cooperativity.” The theoretical advantages of this combination approach, compared to the use of mitochondrial inhibitors alone, are higher therapeutic efficacy and specificity.

### 7.1. Antimetabolic Cooperativity

The antimetabolic cooperativity can be seen as the pharmacological inhibition of several complementary metabolic pathways to elicit a robust elimination of malignant cells. Mitochondria-targeted antioxidants, which have a low toxicity for normal cells, synergize with the antiglycolytic drug, 2-deoxyglucose (2-DG), to kill breast tumor cells* in vitro* and* in vivo* [96]. In addition, the combination of the mitochondrial complex I inhibitor, metformin, and 2-deoxyglucose induces almost complete cytotoxicity in prostate cancer cells without significant death of normal epithelial cells [97]. In line with this, myeloma cells exposed to the FDA-approved GLUT4 inhibitor, ritonavir, benefit from the adjuvant metformin treatment to target compensatory mitochondrial metabolism [98]. Thus, in contrast to a single agent treatment, simultaneous administration of ritonavir (blocking glycolysis) and metformin (inhibiting mitochondrial metabolism) represents a drug combination that could easily be extrapolated to humans to drastically eradicate myeloma cells [98]. In other preclinical models, the halting of tumor growth requires the simultaneous inhibition of mitochondrial biogenesis and glycolysis on top of glutamine use [72]. This result underlies the abundance of alternative metabolic pathways able to compensate for each other. In this context, it would be interesting to develop a robust, efficient screening strategy to identify the relevant metabolic targets' combinations for cancer therapy.

### 7.2. Towards Novel Forms of Antimetabolic Cooperativity by the Combination of Oncogenic Kinase Inhibitors and Mitochondrial Targeting Drugs

Although at first most molecular-targeted drugs demonstrate impressive response rates, patients do relapse over time. One biological reason is that targeted drugs (such as BRAFi), unlike genotoxic agents, do not induce massive cell deaths resulting in the persistence of a drug-tolerant subpopulation of cells, prone to subsequent mutations further increasing resistance (see Figure 4). Among novel potential therapeutic associations, the combination of oncogenic kinase inhibitors (molecular-targeted therapy) with mitochondrial activity inhibitors was proposed to improve neoplasia control and reduce the development of drug resistance (Table 2). This therapeutic combination is also called “synthetic lethality.” The proposed pharmacological combination consists in the following: (i) a* first-hit* inhibition of oncogenic drivers by molecular-targeted drugs (such as BRAFi) deeply affecting the metabolism (“weakened cancer cells”) leading to the inhibition of glycolysis and promoting mitochondrial metabolism (in a drug-tolerant subpopulation of cells, mitochondrial reprogramming allows cells to survive); (ii) mitochondrial addiction rendering the drug-tolerant subpopulation of cells infinitely sensitive to the lethal effects of mitochondrial inhibitors, transforming an apparent disadvantage into a therapeutic advantage (*second-hit*). This two-hit strategy, notably the combination of BRAFi with mitochondrial inhibitors, seems effective in many preclinical models (Table 2). Authors reported that the mitochondrial protein dihydrolipoamide S-acetyltransferase (DLAT), a component of the pyruvate dehydrogenase (PDH) complex, is necessary for Ph^+^ leukemia cells to survive in the presence of BCR-ABL inhibitors [36]. Therefore, the simultaneous blockage of BCRABL (or another tyrosine kinase, FLT3) and inhibition of mitochondria promote cancer cell death. In preclinical models, this new combination kills drug-tolerant subpopulations of cells and thereby minimalizes the risk of relapse. The theoretical advantages attributed to the combination of kinase inhibitors and inhibitors of mitochondrial metabolism include the following: (i) oncogenic kinase inhibitors constrain tumors to use mitochondria, regardless of the initial metabolic heterogeneity of tumor cells; (ii) in combination therapy, the mitochondrial addiction induced by oncogenic kinase inhibitors renders tumor cells “oversensitive” to mitochondrial inhibition. It allows the reduction of mitochondrial inhibitors' doses, thus diminishing the toxicity to healthy tissues and increasing tumor specificity. This hypothesis is corroborated by studies demonstrating the better specificity of oligomycin for cancer cells exposed to oncogenic kinase inhibitors rendered addict to mitochondrial OXPHOS [36].

It is likely that such a combination strategy would be less challenging to implement safely in clinical practice than the use of mitochondrial poisons alone. Regardless of these considerations, the ability of mitochondrial inhibitors to potentiate molecular-targeted therapies requires further preclinical and clinical investigations.

## 8. Conclusion 

Over the last decade, the accumulation of knowledge on the metabolic organization of cancer cells has opened up new avenues for developing realistic approaches to target mitochondrial metabolism. Nevertheless, complex and dynamic metabolic networks constitute challenging hurdles for mitochondrial targeting in cancer therapy. Thus, it seems possible that targeting a single component of the mitochondrial metabolism would be ineffective for anticancer therapy. Conversely, the association of mitochondrial inhibitors to drugs targeting specific compensatory metabolic pathways might represent a promising strategy for cancer treatment. Particularly, recent evidence has underlined that associating inhibitors of oncogenic kinases (which inhibit glycolysis and render cells addict to mitochondrial metabolism) with mitochondrial-targeting drugs could be translated into clinical applications in hope to fight cancer. However, more fundamental and clinical studies are warranted before envisioning mitochondrial metabolism as a valuable target for cancer treatment.

## Figures and Tables

**Table 1:** Examples of potential mitochondrial targets for cancer treatment.

Mitochondrial targets	Drugs (phase of development)	Mechanisms of action	Resulting effects on mitochondrial activity	Anticancer effects	Cancer types	Reference
CPT1 carnitine *O*-palmitoyltransferase 1	Etomoxir (clinical trials)	CPT1 inhibitor: inhibits mitochondrial import of FA	Reduction in FA oxidation and OXPHOS	(i) Reduces viability of leukemia stem cell (ii) Potentiates the effects of chemotherapy	AML	[16]

IDH2 mutant	AGI-6780 (clinical trials)	(R140Q) IDH2 mutant inhibitor: reduces TCA flux (reverse and forward) and lipid biosynthesis	Reduction in the accumulation of the oncometabolite, R-2-hydroxyglutarate (2-HG)	Promotion of the differentiation of leukemic blasts	AML	[17]

Complex V or FO-F1 ATPase	Oligomycin A (preclinical data)	Inhibitor of the FO subunit	(i) Inhibition of ATP synthesis and reduction of electron flux through the ETC (ii) Induction of ROS and MPTP?	(i) Loss of viability (ii) Inhibits the formation of spheroids	Several tumors including breast cancer	[18]

Electron transport chain	Elesclomol (clinical trials)	Inhibitor of the ETC by picking up electrons to the ETC	Inhibition of the electron transport flux and promoting ROS production	Reduction in proliferation and induction of apoptotic cell death	Melanoma including those resistant to BRAF mutant inhibitors	[5, 19, 20]
NADH: ubiquinone oxidoreductase or complex I	(i) Rotenone (preclinical data)	(i) CI inhibitor	Decreases OXPHOS and mitochondrial oxidative metabolism	Kills cancer stem cells (50–100 times more potent in mammospheres than in isolated cells)	Breast cancer	[21]
	(ii) Metformin or phenformin (a biguanide related to metformin) (clinical trials)	(ii) Concentrates into mitochondrial matrix and also possesses systemic effects (diabetes drugs) and also AMPK activators. Phenformin is a more potent mitochondrial inhibitor than metformin	Reduction of oxidative phosphorylation and ATP synthesis	(i) Inhibition of cell proliferation and inducing cell death (ii) Decreasing the risk of cancer	Leukemia and several solid tumors	[22, 23]
Ubiquinol: cytochrome c oxidoreductase or complex III	Phenethyl isothiocyanate (PEITC)	CIII inhibitor	Decreases OXPHOS and induces ROS overproduction	Kills cancer cells	Prostate cancer	[24]
Pyruvate dehydrogenase kinase	Dichloroacetate (DCA) (clinical trials)	PDK isoenzymes inhibitor increases PDH activity (and reduces glycolytic pathways?)	Increase in pyruvate oxidation, OXPHOS, resulting in ROS overproduction		Melanoma, sorafenib resistant hepatocarcinoma, glioblastoma, and other tumors	[5, 25–27]

Glutaminase	(i) 968 (ii) BPTES [*bis*-2-(5-phenyl-acetamido-1,2,4-thiadiazoyl-2-yl)ethyl sulfide] (preclinical data) (iii) CB-839 (preclinical data and clinical trials)	Inhibit mitochondrial glutaminase and conversion of glutamine in glutamate	Reduce the glutamine metabolism in mitochondria	Block cell growth and invasion	Breast cancer, glioblastoma, and other tumors CB-839 is a selective glutaminase inhibitor currently in phase I clinical trials	[28–30]

Mitochondrial translation	Tigecycline (preclinical) and other mitochondrially targeted antibiotics	Antimicrobial inhibits mitochondrial protein translation	Suppress mitochondrial biogenesis and respiration	Loss of viability	Selectively kill AML stem cells Tumor initiating cells from several cancer cell types	[31] [32]

ANT	PENAO (4-(N-(S-penicillaminylacetyl)amino) phenylarsonous acid) (clinical trials)	Inactivate ANT by oxidation of Cys residues	Induce MPTP and ROS	Loss of viability	Breast cancer	[33]

**Table 2:** Prototypic examples of novel anticancer strategies combining oncogenic kinases and mitochondrial activity inhibitors.

Inhibitor of oncogenic driver (target; drug)	Induction of mitochondrial addiction	Mechanism(s) of mitochondrial activation	Combination (target; drug)	Cancer type; cell type; experimental models	Anticancer effects on cells resistant to targeted therapy	Reference
PI3K inhibitor; PX-866	+	Paradoxical activation of mitochondrial Akt2 and thereby of cyclophilin D phosphorylation	Cyclophilin D inhibitor; Gamitrinib	Glioblastoma, breast and lung adenocarcinoma; cell lines and patient organotypic culture; *in vitro* and *in vivo* (xenograft models)	nd	[34]

MEK inhibitor; Selumetinib	+/−	MITF-PGC-1*α* axis regulated by mTORC1/2	mTORC1/2 inhibitor; AZD 8055	Subpopulation of melanoma with high levels of OXPHOS; cell lines and tumor biopsies; *in vitro* and *in vivo* (xenograft models)	**+**	[35]

TKI such as BCRABL inhibitor (imatinib) or FLT3 inhibitor (quirzatinib)	+	Pyruvate entry in the TCA cycle	Oligomycin A (at low nmol/L concentrations)	BCRABL+ CM leukemia FLT3 ITD AML; cell lines; *in vitro* and *in vivo* (mouse leukemia models)	nd	[36]

MEK inhibitor (AZD8330) and dual PI3K/mTOR inhibitor (BEZ235)	+	Mitochondrial biogenesis and activity	Oligomycin A	Pancreatic ductal adenocarcinoma with mutated KRAS; cell lines; *in vitro* and *in vivo* (genetically engineered mouse models)	nd	[37]

Mutated BRAF inhibitor (vemurafenib)	+	PGC-1*α* dependent and independent pathways	Mitochondrial-targeted prooxidative drug, elesclomol -KCN	Melanoma; cell lines and patient biopsies; *in vitro* and *in vivo* (patient-derived tumor xenograft models) cell lines	+ +JARID positive slow cycling melanoma	[19] [38]
	+		Phenformin	Melanoma; cell lines; *in vitro* and *in vivo* (genetically engineered mouse models)	Delay the onset of acquired resistance to BRAFi	[39]
	+	PGC-1*α* dependent mitochondrial biogenesis	Oligomycin A	Melanoma; cell lines and clinical samples; *in vitro* and *in vivo* (xenograft models)	+JARID positive slow cycling melanoma	[40, 41]
	nd	nd	PDK inhibitor (DCA)	Melanoma; cell lines; *In vitro*	nd	[28]
	nd	nd	Metformin	Melanoma patients; retrospective analysis of clinical records	Not significant	[42]

Akt inhibitor	nd	nd	Metformin	Acute myeloid leukemia; cell lines; *in vitro* and *in vivo* (xenograft models)	nd	[22]

Multiple kinase inhibitor (sorafenib)	nd	nd	PDK inhibitor (DCA)	Hepatocarcinoma; cell lines; *in vitro* and *in vivo* (xenograft models)	+	[25]
